# Circulating endothelial signatures correlate with worse outcomes in COVID-19, respiratory failure and ARDS

**DOI:** 10.1186/s13054-025-05596-0

**Published:** 2025-10-14

**Authors:** Ana C. Costa Monteiro, Harry Pickering, Aartik Sarma, Clove S. Taylor, Meagan M. Jenkins, Fei-Man Hsu, Brian Nadel, Ofer Levy, Lindsey R. Baden, Esther Melamed, Lauren I. R. Ehrlich, Grace A. McComsey, Rafick P. Sekaly, Charles B. Cairns, Elias K. Haddad, Albert C. Shaw, David A. Hafler, Ruth R. Montgomery, David B. Corry, Farrah Kheradmand, Mark A. Atkinson, Scott C. Brakenridge, Nelson I. Agudelo Higuita, Jordan P. Metcalf, Catherine L. Hough, William B. Messer, Bali Pulendran, Kari C. Nadeau, Mark M. Davis, Linda N. Geng, Ana Fernandez-Sesma, Viviana Simon, Florian Krammer, Monica Kraft, Chris Bime, Carolyn S. Calfee, David J. Erle, Steve Bosinger, Walter Eckalbar, Holden Maecker, Adeeb Rahman, Leying Guan, Bjoern Peters, Steven H. Kleinstein, Alison D. Augustine, Joann Diray-Arce, Patrice M. Becker, Nadine Rouphael, IMPACC Network, Patrice M. Becker, Alison D. Augustine, Steven M. Holland, Lindsey B. Rosen, Serena Lee, Tatyana Vaysman, Al Ozonoff, Joann Diray-Arce, Jing Chen, Alvin Kho, Carly E. Milliren, Annmarie Hoch, Ana C. Chang, Kerry McEnaney, Brenda Barton, Claudia Lentucci, Maimouna D. Murphy, Mehmet Saluvan, Tanzia Shaheen, Shanshan Liu, Caitlin Syphurs, Marisa Albert, Arash Nemati Hayati, Robert Bryant, James Abraham, Sanya Thomas, Mitchell Cooney, Meagan Karoly, Matthew C. Altman, Naresh Doni Jayavelu, Scott Presnell, Bernard Kohr, Tomasz Jancsyk, Azlann Arnett, Bjoern Peters, James A. Overton, Randi Vita, Kerstin Westendorf, James A. Overton, Ofer Levy, Hanno Steen, Patrick van Zalm, Benoit Fatou, Kinga K. Smolen, Arthur Viode, Simon van Haren, Meenakshi Jha, David Stevenson, Lindsey R. Baden, Kevin Mendez, Jessica Lasky-Su, Alexandra Tong, Rebecca Rooks, Michael Desjardins, Amy C. Sherman, Stephen R. Walsh, Xhoi Mitre, Jessica Cauley, Xiofang Li, Bethany Evans, Christina Montesano, Jose Humberto Licona, Jonathan Krauss, Nicholas C. Issa, Jun Bai Park Chang, Natalie Izaguirre, Scott R. Hutton, Greg Michelotti, Kari Wong, Scott J. Tebbutt, Casey P. Shannon, Rafick-Pierre Sekaly, Slim Fourati, Grace A. McComsey, Paul Harris, Scott Sieg, George Yendewa, Mary Consolo, Heather Tribout, Susan Pereira Ribeiro, Charles B. Cairns, Elias K. Haddad, Michele A. Kutzler, Mariana Bernui, Gina Cusimano, Jennifer Connors, Kyra Woloszczuk, David Joyner, Carolyn Edwards, Edward Lee, Edward Lin, Nataliya Melnyk, Debra L. Powell, James N. Kim, I. Michael Goonewardene, Brent Simmons, Cecilia M. Smith, Mark Martens, Brett Croen, Nicholas C. Semenza, Mathew R. Bell, Sara Furukawa, Renee McLin, George P. Tegos, Brandon Rogowski, Nathan Mege, Kristen Ulring, Pam Schearer, Judie Sheidy, Crystal Nagle, Vicki Seyfert-Margolis, Nadine Rouphael, Steven E. Bosinger, Arun K. Boddapati, Greg K. Tharp, Sonia Wimalasena, Kathryn L. Pellegrini, Brandi Johnson, Bernadine Panganiban, Christopher Huerta, Evan J. Anderson, Hady Samaha, Jonathan E. Sevransky, Laurel Bristow, Elizabeth Beagle, David Cowan, Sydney Hamilton, Thomas Hodder, Amer Bechnak, Andrew Cheng, Aneesh Mehta, Caroline R. Ciric, Christine Spainhour, Erin Carter, Erin M. Scherer, Jacob Usher, Kieffer Hellmeister, Laila Hussaini, Lauren Hewitt, Nina Mcnair, Ana Fernandez-Sesma, Viviana Simon, Florian Krammer, Harm Van Bakel, Seunghee Kim-Schulze, Ana Silvia Gonzalez-Reiche, Jingjing Qi, Brian Lee, Juan Manuel Carreño, Gagandeep Singh, Ariel Raskin, Johnstone Tcheou, Zain Khalil, Adriana van de Guchte, Keith Farrugia, Zenab Khan, Geoffrey Kelly, Komal Srivastava, Lily Q. Eaker, Maria C. Bermúdez-González, Lubbertus C. F. Mulder, Katherine F. Beach, Miti Saksena, Deena Altman, Erna Kojic, Levy A. Sominsky, Arman Azad, Dominika Bielak, Hisaaki Kawabata, Temima Yellin, Miriam Fried, Leeba Sullivan, Sara Morris, Giulio Kleiner, Daniel Stadlbauer, Jayeeta Dutta, Hui Xie, Manishkumar Patel, Kai Nie, Adeeb Rahman, William B. Messer, Catherine L. Hough, Sarah A. R. Siegel, Peter E. Sullivan, Zhengchun Lu, Amanda E. Brunton, Matthew Strand, Zoe L. Lyski, Felicity J. Coulter, Courtney Micheleti, Holden Maecker, Bali Pulendran, Kari C. Nadeau, Yael Rosenberg-Hasson, Michael Leipold, Natalia Sigal, Angela Rogers, Andrea Fernandes, Monali Manohar, Evan Do, Iris Chang, Alexandra S. Lee, Catherine Blish, Henna Naz Din, Jonasel Roque, Linda N. Geng, Maja Artandi, Mark M. Davis, Neera Ahuja, Samuel S. Yang, Sharon Chinthrajah, Thomas Hagan, Elaine F. Reed, Joanna Schaenman, Ramin Salehi-Rad, Adreanne M. Rivera, Harry C. Pickering, Subha Sen, David Elashoff, Dawn C. Ward, Jenny Brook, Estefania Ramires-Sanchez, Megan Llamas, Claudia Perdomo, Clara E. Magyar, Jennifer Fulcher, David J. Erle, Carolyn S. Calfee, Carolyn M. Hendrickson, Kirsten N. Kangelaris, Viet Nguyen, Deanna Lee, Suzanna Chak, Rajani Ghale, Ana Gonzalez, Alejandra Jauregui, Carolyn Leroux, Luz Torres Altamirano, Ahmad Sadeed Rashid, Andrew Willmore, Prescott G. Woodruff, Matthew F. Krummel, Sidney Carrillo, Alyssa Ward, Charles R. Langelier, Ravi Patel, Michael Wilson, Ravi Dandekar, Bonny Alvarenga, Jayant Rajan, Walter Eckalbar, Andrew W. Schroeder, Gabriela K. Fragiadakis, Alexandra Tsitsiklis, Eran Mick, Yanedth Sanchez Guerrero, Christina Love, Lenka Maliskova, Michael Adkisson, Aleksandra Leligdowicz, Alexander Beagle, Arjun Rao, Austin Sigman, Bushra Samad, Cindy Curiel, Cole Shaw, Gayelan Tietje-Ulrich, Jeff Milush, Jonathan Singer, Joshua J. Vasquez, Kevin Tang, Legna Betancourt, Lekshmi Santhosh, Logan Pierce, Maria Tecero Paz, Michael Matthay, Neeta Thakur, Nicklaus Rodriguez, Nicole Sutter, Norman Jones, Pratik Sinha, Priya Prasad, Raphael Lota, Sadeed Rashid, Saurabh Asthana, Sharvari Bhide, Tasha Lea, Yumiko Abe-Jones, David A. Hafler, Ruth R. Montgomery, Albert C. Shaw, Steven H. Kleinstein, Jeremy P. Gygi, Shrikant Pawar, Anna Konstorum, Ernie Chen, Chris Cotsapas, Xiaomei Wang, Leqi Xu, Charles Dela Cruz, Akiko Iwasaki, Subhasis Mohanty, Allison Nelson, Yujiao Zhao, Shelli Farhadian, Hiromitsu Asashima, Omkar Chaudhary, Andreas Coppi, John Fournier, M. Catherine Muenker, Allison Nelson, Khadir Raddassi, Michael Rainone, William Ruff, Syim Salahuddin, Wade L. Shulz, Pavithra Vijayakumar, Haowei Wang, Esio Wunder, H. Patrick Young, Albert I. Ko, Xiomei Wang, Denise Esserman, Leying Guan, Anderson Brito, Jessica Rothman, Nathan D. Grubaugh, David B. Corry, Farrah Kheradmand, Li-Zhen Song, Ebony Nelson, Jordan P. Metcalf, Nelson I. Agudelo Higuita, Lauren A. Sinko, J. Leland Booth, Douglas A. Drevets, Brent R. Brown, Monica Kraft, Chris Bime, Jarrod Mosier, Heidi Erickson, Ron Schunk, Hiroki Kimura, Michelle Conway, Dave Francisco, Allyson Molzahn, Connie Cathleen Wilson, Ron Schunk, Trina Hughes, Bianca Sierra, Mark A. Atkinson, Scott C. Brakenridge, Ricardo F. Ungaro, Brittany Roth Manning, Lyle Moldawer, Jordan Oberhaus, Faheem W. Guirgis, Brittney Borresen, Matthew L. Anderson, Lauren I. R. Ehrlich, Esther Melamed, Cole Maguire, Dennis Wylie, Justin F. Rousseau, Kerin C. Hurley, Janelle N. Geltman, Nadia Siles, Jacob E. Rogers, Pablo Guaman Tipan, CAFPINT Site investigators of the PALISI Network, Michael Agus, Vijay Srinivasan, Ranjit S. Chima, Neal J. Thomas, Simon Li, Alan Pinto, Christopher Newth, Amanda B. Hassinger, Kris Bysani, Edward Vincent Faustino, Eliotte Hirshberg, Kupper Wintergerst, Janice E. Sullivan, Adam Schwarz, Lauren Sorce, Lauren Marsillio, Natalie Cvijanovich, Heidi Flori, Nga Pham, Mary Dahmer, Myke Federman, Kayley Wong, Sitaram S. Vangala, Matteo Pellegrini, Brunilda Balliu, Kinisha P. Gala, Sholeen Nett, Marcy Singleton, Neethi Pinto, Grace Chong, Shirley Viteri, Anil Sapru, Patrick McQuillen, Matt Zinter, Michael Agus, Hrishikesh Kulkarni, Joanna M. Schaenmann, Ramin Salehi-Rad, Michael A. Matthay, Elaine F. Reed, Anil Sapru

**Affiliations:** 1 University of California School of Medicine- Los Angeles, Los Angeles, CA USA; 2 University of California- San Francisco, San Francisco, CA USA; 3 University of Southern California, Los Angeles, CA USA; 4 Department of Pediatrics, Boston Children’s Hospital, Boston, MA USA; 5 Harvard Medical School, Boston, MA USA; 6 Broad Institute of MIT & Harvard, Boston, MA USA; 7 University of Texas- Austin, Austin, TX USA; 8 Case Western Reserve University and University Hospitals of Cleveland, Cleveland, OH USA; 9 Emory University, Atlanta, GA USA; 10 Drexel University College of Medicine, Philadelphia, PA USA; 11 Yale University School of Medicine, New Haven, CT USA; 12 Baylor School of Medicine, Houston, TX USA; 13 University of Florida College of Medicine, Gainesville, FL USA; 14 University of Washington School of Medicine, Seattle, WA USA; 15 Oklahoma University Health Sciences Center, Oklahoma City, OK USA; 16 Oregon Health & Science University, Portland, OR USA; 17 Stanford University School of Medicine, Stanford, CA USA; 18 Harvard T. H. Chan School of Public Health, Boston, MA USA; 19 Icahn School of Medicine at Mount Sinai, New York, NY USA; 20 University of Arizona College of Medicine, Phoenix, AZ USA; 21 Immunai, New York, NY USA; 22 La Jolla Institute for Allergy and Immunology, La Jolla, CA USA; 23 National Institute of Allergy and Infectious Disease, Bethesda, USA; 24 Veteran Affairs- Greater Los Angeles, Los Angeles, CA USA

## Abstract

**Background:**

Elevated circulating endothelial cells (CECs), released from monolayers after insult, have been implicated in worse outcomes in ARDS and COVID-19, however there is no consensus proteomic phenotype that define CECs. We queried whether a transcriptomic approach would alternatively support the presence of endothelial cells in circulation and correlate with worsening respiratory failure.

**Methods:**

To test whether elevated endothelial cell signatures (ECS) in circulation plays a role in worse respiratory outcomes, we used unsupervised bulk-transcriptome deconvolution to quantify ECS% in two cohorts. Our pilot analysis included pediatric patients requiring invasive mechanical ventilation (CAF-PINT, NCT01892969). Our validation cohort included adult hospitalized patients with COVID-19 (IMPACC, NCT04378777), testing the association of ECS% to outcomes in patients at risk of acute respiratory failure/ARDS. Primary outcome was 28-day mortality.

**Results:**

In CAF-PINT, day 0 ECS% was higher in non-survivors compared to survivors of respiratory failure (2.8%, IQR 2.4–3.4% versus 2.6%, IQR 2.2–3.0% n = 244, p < 0.05, Wilcoxon rank-sum). In IMPACC, baseline ECS% (< 72 h of hospitalization) was higher in COVID-19 non-survivors versus survivors (2.9%, IQR 2.6–3.4%, versus 2.7%, IQR 2.3–3.1%, n = 932, p < 0.001, Wilcoxon rank-sum). Each 1% increase in baseline ECS% was significantly associated with mortality (adjusted OR 1.36, CI 1.03–1.79) by multivariable logistic regression. Increased baseline ECS% was associated with worse respiratory trajectories (2.5%, IQR 2.2–2.8% for trajectory with no oxygen requirements, 2.9%, IQR 2.6–3.4% for the trajectory with fatal outcome by day 28, n = 932, p < 0.001, one-way ANOVA).

**Conclusion:**

Quantifying ECS by deconvolution supports a transcriptomics-driven approach towards the non-invasive evaluation of endothelial damage in respiratory outcomes. This is a first step towards elucidating mechanistic components linking endothelial damage to ARDS utilizing non-invasive, circulating transcriptomic data by leveraging a novel deconvolution approach.

**Supplementary Information:**

The online version contains supplementary material available at 10.1186/s13054-025-05596-0.

## Introduction

Acute respiratory distress syndrome (ARDS) is a heterogeneous condition with high mortality and limited therapies [1–3]. The identification of varied circulating biomarkers that are associated with worse outcomes may enable the predictive enrichment necessary for discovery of novel therapeutic strategies. Endothelial damage is an early and critical event in the pathogenesis of pediatric and adult acute lung injury. Elevated circulating endothelial cells (CECs) are released from monolayers after endothelial insult and have been described as early as the 1970s [4, 5] to correlate with disease severity in conditions like myocardial infarction, malignancy, and vasculitis [6–10]. CECs have also been associated with worse outcomes in smaller studies of ARDS and COVID-19 [11, 12].

There is no established gold standard protocol for identifying CECs from circulation. Recently, the identification and enumeration of CECs has relied on multichannel flow cytometry [11–15], but disagreement on how CECs are phenotypically defined, and the varying methods for staining and gating the population of interest mitigates the utility of CEC enumeration as a tool for understanding the role of endothelial damage in ARDS pathogenesis or as a marker of ARDS prognosis [14, 16, 17]. In turn, approaches that target the transcriptomic signature of endothelial cells in circulation may provide a surrogate measure of endothelial damage from blood samples. We used an unsupervised, bulk-transcriptome based deconvolution method to quantify endothelial signatures from peripheral blood by leveraging pilot (CAF-PINT; Coagulation and Fibrinolysis in Pediatric Insulin Titration) [18] and validation (IMPACC; Immunophenotyping Assessment in a COVID-19 Cohort) [19] multisite clinical cohorts to ask whether the relative abundance of the endothelial cell signature (ECS) in circulation was associated with worse outcomes in patients with/at risk for respiratory failure and ARDS. Using this approach, we found that the abundance of the ECS correlates with worse outcomes in children and adults at risk for and with ARDS.

## Methods

### Patient populations

We utilized two cohorts. The pilot analysis utilized a cohort of pediatric patients receiving invasive mechanical ventilation within 96 h before randomization to insulin titration to achieve lower (80–110 mg/dL) or higher (150–180 mg/dL) blood glucose targets (CAF-PINT, NCT01892969, a co-enrolled subgroup of HALF-PINT, NTC01565941, n = 244 at first visit) [18, 20]. Clinical data and samples for CAF-PINT were collected at hospital days 0, 2, and 4 after randomization. The validation cohort included hospitalized adult patients with COVID-19 who had RNA sequencing data available for the first visit (IMPACC, NCT04378777, n = 932 at first visit) [19]. Clinical data and samples for IMPACC were collected at enrollment (< 72 h) and at days 4, 7, 14, 21, and 28 while participants were hospitalized. Biologic samples were also collected at 3-month intervals up to 12 months after hospital discharge. For both cohorts, we had clinical mortality data through day 28.

### Deconvolution algorithm

We used an unsupervised, bulk-transcriptome based deconvolution method to quantify endothelial cell signatures (ECS) from bulk RNA of peripheral blood named GEDIT [21, 22] (The Gene Expression Deconvolution Interactive Tool) to perform the deconvolution analysis with the bulk RNASeq data from two independent reference sets (below) as input. The deconvolution algorithm identifies “signature genes”, which are the top 50 genes for each cell type, as derived from the reference set that is common to the experimental cohort, and then restricts the deconvolution analysis to the genes that are included in at least one cell type. The algorithm then incorporates all the expressed gene data from the experimental cohort by using non-negative least squares-based deconvolution to determine the most likely cell composition that would add up to the measured expression level for each gene. This approach finds the optimal solution to determine the most likely total cell composition for a particular sample. The two reference sets utilized were:

1. The Human Primary Cell Atlas (HPCA) [23]- An atlas of human primary cells based upon the meta-analysis of gene expression data derived from microarray datasets from 745 samples. Using the HPCA, GEDIT identified 50 signature endothelial cell genes (supplemental files).
2. The Human Lung Cell Atlas (HLCA), a single-cell transcriptomic atlas of the human respiratory system encompassing data from 486 donors over 49 datasets [24], was used as the alternate, or validation, reference set for deconvolution. Using the HLCA, GEDIT identified 50 signature endothelial cell genes (supplemental files)

### Selected outcomes and variables

Primary outcome was 28-day mortality; secondary outcomes were 28-day disease trajectory based on longitudinal clustering of a respiratory ordinal score (IMPACC) [25] or ARDS status at any point during hospitalization (CAF-PINT). Adjustment variables were selected a priori according to clinical relevance and included demographics (race, ethnicity, age, sex), and BMI; for disease severity indices, IMPACC included SOFA score and CAF-PINT utilized PRISM-III. Finally, CAF-PINT included randomization to low or high glucose targets. Missing data were not imputed.

### Statistics

Continuous variables are presented as median (IQR). Categorical variables are presented as n (%). Student’s t-test was used to test for a statistical difference between two continuous variables that are approximated by a normal distribution. The Wilcoxon rank-sum test was used for statistical differences between two continuous variables with skewed distribution. A chi-squared test for proportions tested statistical differences between frequencies of categorical variables. We utilized one-way ANOVA (normally distributed data) or Kruskal–Wallis (non parametric data) to test statistical significance for differences between multiple groups. Pearson and Spearman correlation were used to test the linear relationship between two continuous variables. We plotted a receiver operating curve to calculate the area under the curve between ECS% and mortality and PARDS outcomes in CAFPINT. We also ran Hosmer–Lemeshow (HL) tests to evaluate the strength of the above relationships. For HL tests, we categorized ECS% into the largest number of bins permissible for the model (14 bins).

Univariable and multivariable logistic regression were used to estimate and test the relationship between variables and primary and secondary outcomes. The interaction between ECS% and disease severity, age or blood glucose target (latter exclusively in CAF-PINT), were tested in separate logistic regression models for 28-day mortality based on differences noted in Tables 1 and 2.

**Table 1:** Demographic and medical characteristics of CAF-PINT patients

	Survivors (N = 212)	Non-survivors (N = 32)	p-value
Age (Years)			
Median [IQR]	6.4 [2.1,12.5]	6.4 [2.4,14.1]	NS
Sex			
Female	103 (48.6%)	18 (56.3%)	NS
Male	109 (51.4%)	14 (43.8%)	
Race			
American Indian/Alaska Native	2 (0.9%)	0 (0%)	NS
Asian	9 (4.2%)	2 (6.3%)	
Black/African American	53 (25.0%)	7 (21.9%)	
Multiple	9 (4.2%)	1 (3.1%)	
Native Hawaiian/Pacific Islander	1 (0.5%)	0 (0%)	
Unknown/Decline to Answer	4 (1.8%)	0 (0%)	
White	134 (63.2%)	22 (68.8%)	
Ethnicity			
Hispanic or Latino	50 (23.6%)	8 (25.0%)	NS
Not Hispanic or Latino	162 (76.4%)	24 (75.0%)	
Hours of Intubation Pre-Randomization			
Median [IQR]	30.0 [20.0,45.0]	26.5 [21.3,41.0]	NS
Blood Glucose Target Randomization Arm			
Low Glucose Target	101 (47.6%)	15 (46.9%)	NS
High Glucose Target	111 (52.4%)	17 (53.1%)	
PRISM Score			
Median [IQR]	11.0 [5.0,17.0]	15.0 [11.8,25.5]	< 0.01
Any PARDS			
No PARDS	34 (16.0%)	4 (12.5%)	NS
Any PARDS	178 (84.0%)	28 (87.5%)	

PRISM Pediatric Risk of Mortality; PARDS, Pediatric Acute Respiratory Distress Syndrome

**Table 2:** Demographic and medical characteristics of IMPACC patients

	Survivors (N = 839)	Non-Survivors (N = 93)	p-value
Age			
Median [IQR]	58.0 [48.0,68.0]	70.0 [59.0,76.0]	< 0.001
Sex			
Female	341 (40.6%)	29 (31.2%)	NS
Male	498 (59.4%)	64 (68.8%)	
Race			
American Indian / Alaska Native	10 (1.2%)	0 (0%)	NS
Asian	35 (4.2%)	4 (4.3%)	
Black / African American	201 (24.0%)	14 (15.1%)	
Multiple	9 (1.1%)	1 (1.1%)	
Native Hawaiian / Pacific Islander	10 (1.2%)	1 (1.1%)	
Other / Declined	148 (17.6%)	9 (9.7%)	
Unknown / Unavailable	29 (3.5%)	4 (4.3%)	
White	397 (47.3%)	60 (64.5%)	
Ethnicity			
Hispanic or Latino	246 (29.3%)	24 (25.8%)	NS
Not Hispanic or Latino	562 (67.0%)	62 (66.7%)	
Not Specified	31 (3.7%)	7 (7.5%)	
SOFA score at enrollment			
Median [IQR]	1.00 [0, 3.0]	4.00 [1, 9.0]	< 0.001
Missing	25 (3.0%)	2 (2.2%)	
Respiratory status at enrollment			
No supplemental oxygen	212 (25.3%)	10 (10.8%)	< 0.001
Supplemental oxygen required	404 (48.2%)	18 (19.4%)	
NIV or HFNC required	141 (16.8%)	31 (33.3%)	
IMV or ECMO	82 (9.8%)	34 (36.6%)	

NIV, non-invasive ventilation; HFNC, high-flow nasal cannula; IMV, Invasive mechanical ventilation; ECMO, extracorporeal membrane oxygenation; SOFA, Sequential Organ Failure Assessment

A two-sided p-value of 0.05 was considered statistically significant for all analyses. We did not adjust for multiple comparisons. We performed all analyses on R Studio Version 4.2.3 software.

### Bulk RNA sequencing

Peripheral blood mononuclear cell (PBMC) purification and bulk RNA sequencing were performed as previously described [19, 22, 26]. Procedural details can be found in supplemental materials.

### Blood CyTOF

Whole blood staining and acquisition was as previously described [19, 26]. Briefly, samples were stained with a cocktail of 43 antibodies, acquired on the Fluidigm Helios mass cytometer and normalized and concatenated using Fluidigm’s CyTOF software. After removal of multiplets, cells were manually annotated into cell types and cell population count matrices were converted into cell frequencies.

### Ethics

Detailed in supplemental materials.

## Results

### Two demographically and clinically disparate cohorts of patients with or at risk for respiratory failure and ARDS

We compared baseline characteristics between survivors and non-survivors of two cohorts. The pilot analysis utilized CAF-PINT, a multi-center, randomized clinical trial comprised of pediatric patients requiring invasive mechanical ventilation in the intensive care unit randomized to a higher vs. lower blood glucose target [18, 20]. We excluded patients who were intubated for more than 96 h before randomization. In CAF-PINT, 84% had ARDS at any point and 13% were non-survivors by post enrollment day 28 (Table 1). Baseline demographics, duration of invasive mechanical ventilation pre-randomization, or randomization to either blood glucose target strategy were not different between survivors and non-survivors, but baseline severity score (PRISM-III) was significantly different between mortality outcomes (Table 1). The validation cohort, IMPACC, is a multicenter observational cohort of adult patients who were admitted with a diagnosis of COVID-19 to the 20 participating hospitals (n = 932). In IMPACC, 11.5% were intubated, 12.4% were receiving invasive mechanical ventilation (IMV) or ECMO, and 10% were non-survivors by day 28 post enrollment. In IMPACC, there was no data collected on ARDS status. There was a statistically significant difference in age between survivors and non-survivors of IMPACC (Median 59, IQR: 49–67 for survivors vs. median 70, IQR: 59–76 for non-survivors, p < 0.001, Table 2) but there were no observed differences between groups for other baseline demographics. Like CAF-PINT, there were significant differences in baseline severity score (Median SOFA 1.0, IQR: 0.0–3.0 for survivors vs. 4.0 IQR 1.0–9.0 for non-survivors, p < 0.001, Table 2) and ventilation requirements (27% of survivors vs. 70% of non-survivors requiring NIV, HFNC, IMV or ECMO on enrollment, p < 0.001) between survivors and non-survivors at 28 days.

### Endothelial cell signature abundance was associated with worsening respiratory failure

**Fig. 1:**
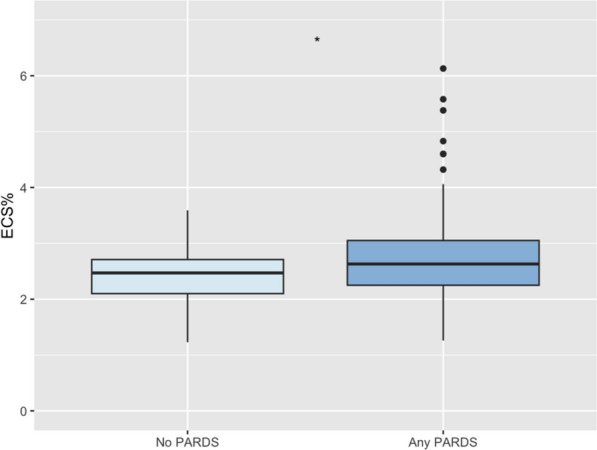
Comparison of percent endothelial cell signature from PBMC population at day 0 between those who developed ARDS versus those that did not develop ARDS at any time point during CAF-PINT study. Pairwise comparison, Wilcoxon rank-sum. *, p < 0.05 (n = 244)

We asked whether ECS% recorded at the earliest time point was associated with worsening oxygen requirements (IMPACC) or prevalence of ARDS (CAFPINT). Indeed, baseline (Day 0) ECS% was higher in patients who developed pediatric ARDS (PARDS) at any time during the CAF-PINT study (median 2.5% IQR 2.1–2.8% for non-PARDS vs. median 2.6% IQR 2.3–3.1% for PARDS, p < 0.05 by Wilcoxon rank-sum; n = 244, Fig. 1). The Hosmer–Lemeshow test revealed a p = 0.8 for the association of ECS% and mortality in CAFPINT, suggesting the association was not solely due to large sample size, and an AUC of 0.6.

ECS% was also significantly associated with development of PARDS in univariable and multivariable analyses after adjustment for demographics and PRISM score (Table S1).

**Fig. 2:**
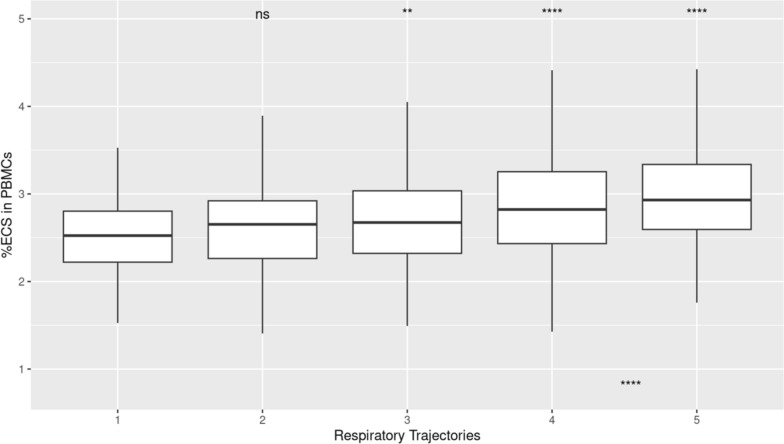
Higher percentage of EC signatures were associated with worse respiratory trajectories of the IMPACC cohort. A modified ordinal score for oxygen requirements, based on the WHO scale, was recorded for all patients at all study timepoints. Based on these ordinal scales, five respiratory trajectories were previously described (Ozonoff et al.) as represented in the x-axis. ECS % as determined by deconvolution of PBMCs collected < 72 h tended to be higher in patients who developed worsening respiratory trajectories over the study period (median 2.53%, IQR 2.22–2.84% for trajectory 1 with minimal requirements, 2.94%, IQR 2.62–3.43% for trajectory 5, defined by fatal outcome by day 28. Overall comparison for differences over all study days, One-way ANOVA, ****, p < 0.0001; Pair-wise comparisons with trajectory 1 as reference, Wilcoxon rank-sum, above each time point. “NS”, Not significant; **, p < 0.01, ****, p < 0.0001 n = 932)

While ARDS status was not recorded in the IMPACC cohort, a modified ordinal score for oxygen requirements, based on the WHO scale, was recorded for all patients at all study timepoints. Based on these ordinal scales, five respiratory trajectories were previously defined [25]. Based on the respiratory trajectory outcome, higher ECS% was associated with worse respiratory trajectories of the IMPACC cohort (median 2.5%, IQR 2.2–2.8% for trajectory with minimal requirements, with statistically significant increases for each trajectory group 2, 3 and 4; 2.9%, IQR 2.6–3.4% for the trajectory 5 with fatal outcome by day 28, p < 0.001, n = 932, one-way ANOVA, Fig. 2).

### Endothelial cell signature abundance was associated with increased mortality

**Fig. 3:**
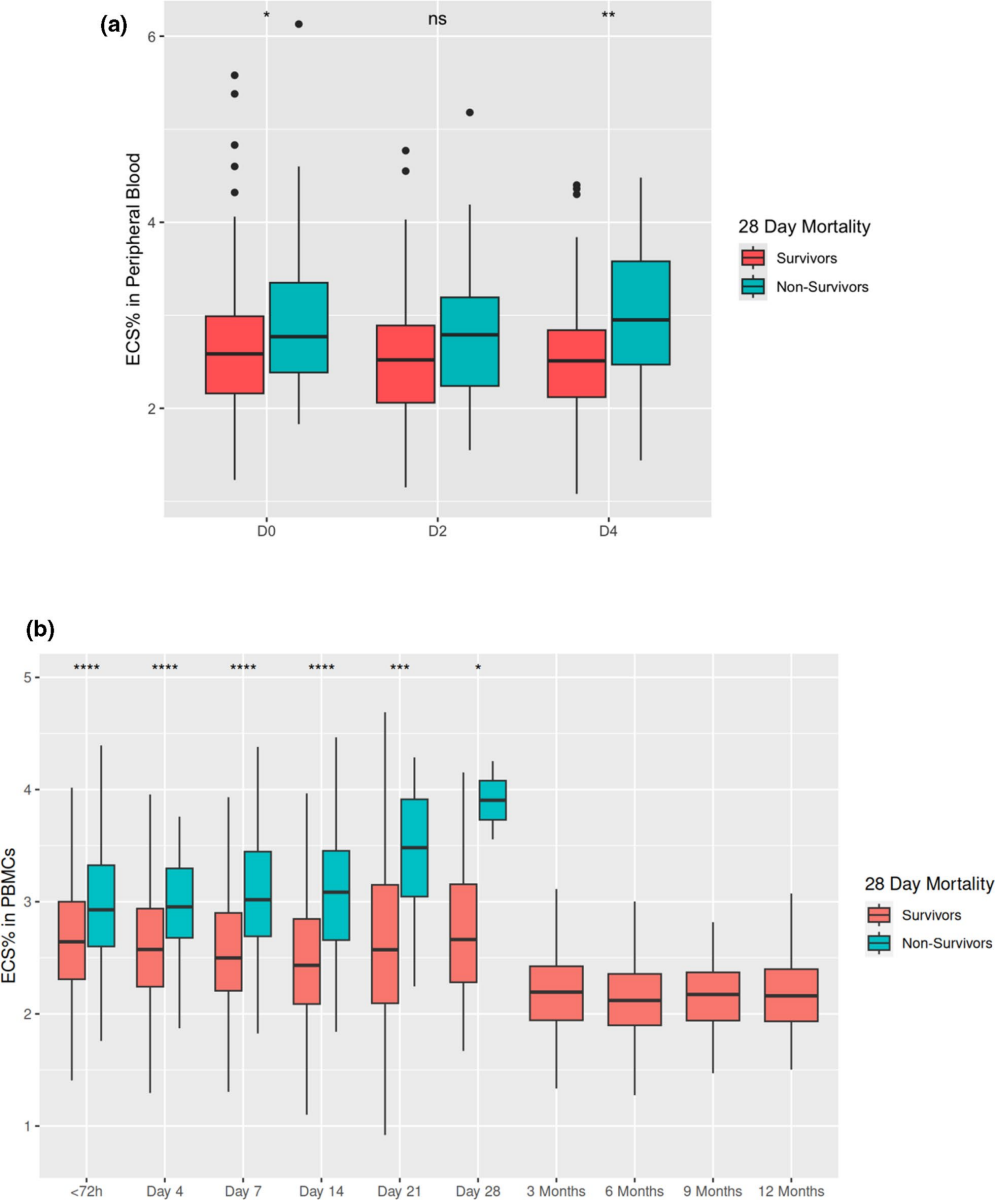
**a** Comparison of percent endothelial cell signature from PBMC population between survivors and non-survivors from CAF-PINT participants who were intubated < 96 h of enrollment, over all collected samples by study day. (Pairwise comparisons between survivors and non-survivors in each time point, Wilcoxon rank-sum. *, p < 0.05; **, p < 0.01; ns, not significant). **b** Comparison of percent endothelial cell signature from PBMC population between survivors and non-survivors from IMPACC participants over all collected samples by study day. Pairwise comparisons between survivors and non-survivors in each time point, Wilcoxon rank-sum. *, p < 0.05; **, p < 0.01; ***, p < 0.001; ****, p < 0.0001

We queried the association of the ECS abundance with mortality. ECS abundance was significantly higher in non-survivors compared to survivors in both cohorts (For CAF-PINT, 2.8% IQR 2.4–3.4% for non survivors vs. 2.6% IQR 2.2–3.0% for survivors on day 0, p < 0.05 Fig. 3a; for IMPACC, 2.9% IQR 2.6–3.4% for non-survivors vs. 2.7% IQR 2.3–3.1% for survivors at < 72 h, p < 0.0001; Fig. 3b). For CAFPINT, the Hosmer–Lemeshow test revealed a P = 0.6, while the AUC was 0.6 for the same association. For IMPACC, the Hosmer–Lemeshow test revealed a P = 0.2, while the AUC was 0.6 for the same association. The ECS% of IMPACC survivors at post discharge time points (Months 3–12) were lower than the ECS% of survivors and non-survivors during the first 28 days post study enrollment (Fig. 3b).

We asked whether ECS% at the earliest recorded time point was associated with mortality by logistic regression. In the CAF-PINT and IMPACC cohorts, percentage increases in the ECS at the first recorded time point were associated with higher odds of 28-day mortality by univariate and multivariate logistic regression. For CAF-PINT, each 1% increase in baseline ECS was significantly associated with 28-day mortality with univariate regression (OR 1.80, CI 1.13–2.92, Table 3). Given that there was a significant difference in PRISM scores between survivors and non-survivors, and the possibility that endothelial shedding/damage may also be affected by baseline disease severity, we tested whether there was an interaction effect between baseline ECS% and PRISM score for the outcome of 28-day mortality, but no significant interaction was found (OR 1.01, CI 0.96–1.08, p = 0.48). Given CAF-PINT participants were randomized to one of two blood glucose targets, and the concern that differences in blood glucose may affect endothelial shedding/damage, we tested whether there was an interaction between ECS abundance and randomization groups. Again, we found no significant interaction between randomization to glucose target and ECS% for the outcome of 28-day mortality (OR 1.44, CI 0.53–4.11, p = 0.48). Baseline ECS% was linearly associated with 28-day mortality after adjustment for demographics, age, PRISM score and randomization to glucose by multivariable logistic regression (OR 1.55, CI 0.91–2.61, Table 3). For IMPACC, each 1% increase in ECS was significantly associated with 28-day mortality with univariate regression (OR 1.48, CI 1.18–1.84, Table 4) and after adjusting for demographics, SOFA score and BMI by multivariable logistic regression (OR 1.36, CI 1.03–1.79, Table 4). Given that there was a significant difference in age and SOFA scores between survivors and non-survivors of the IMPACC trial, we tested whether there was an interaction effect between baseline ECS% and age or baseline SOFA score for the outcome of 28-day mortality, as both age and baseline disease severity could potentially affect ECS abundance and mortality, but no significant interaction was found (p = 0.8 for the interaction of ECS% and SOFA, p = 0.4 for the interaction of ECS% and age, both for mortality outcome).

**Table 3:** CAFPINT 28-day mortality

	OR	CI	p value
Univariable (no adjustments)			
ECS %	1.80	1.132–2.92	< 0.05
Multivariable (adjusted for sex, race and ethnicity)			
ECS %	1.55	0.91–2.61	0.10
Glucose Target Strategy	0.96	0.43–2.13	0.92
PRISM Score	1.07	1.03–1.12	< 0.001
Age (Years)	1.00	0.93–1.08	0.93

**Table 4:** IMPACC 28-day mortality

	OR	CI	p value
Univariable (no adjustments)			
ECS %	1.48	1.18–1.84	< 0.001
Multivariable (adjusted for sex, race and ethnicity)			
ECS %	1.36	1.03–1.79	< 0.05
SOFA Score	1.26	1.19–1.35	< 0.001
Age (Years)	1.06	1.04- 1.09	< 0.001
BMI	1.03	0.998–1.07	0.07

### Validation of deconvolution measurements in the IMPACC study

**Fig. 4:**
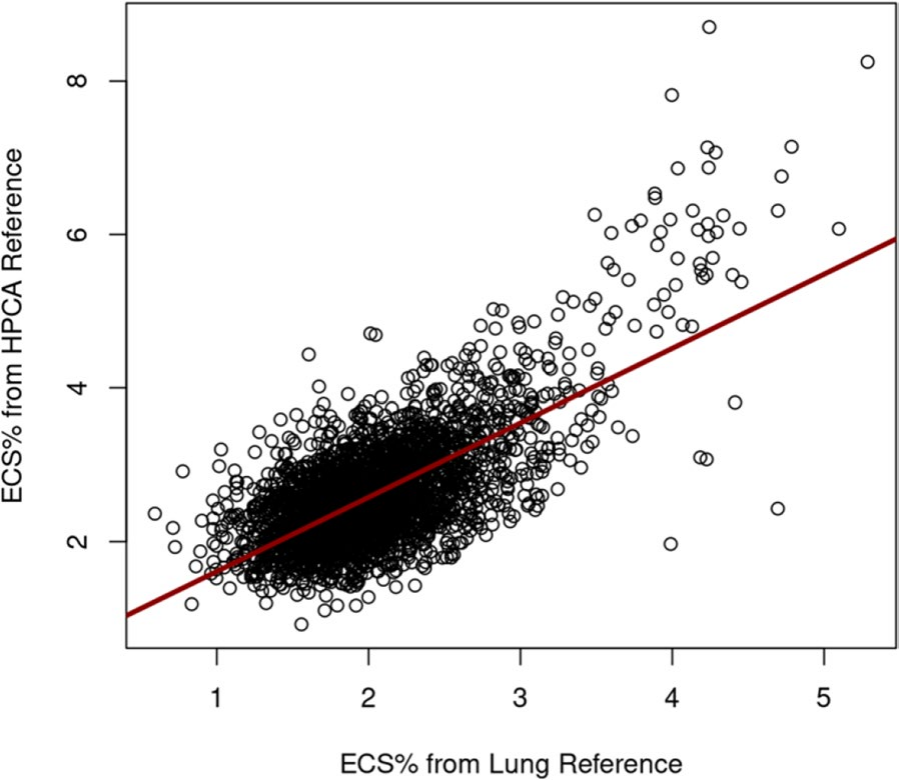
Correlation between abundance of endothelial cell signature in PBMC, expressed as percentage, derived by deconvolution between two different reference sets- the human protein atlas (HPCA) against the human lung cell atlas (HLCA). Pearson correlation 0.69, CI 0.67–0.71, p < 0.0001, n = Pearson correlation 0.69, CI 0.67–0.71, p < 0.0001 n = 2993 (all time points)

We used the IMPACC cohort to cross-validate our deconvolution output in two ways. We first tested whether the results observed were dependent on the choice of reference dataset used for deconvolution. For the analysis previously described, we used the HPCA dataset [27] as it included the most comprehensive list of cells expected to be in circulation. We then queried whether these findings would be replicated when utilizing the Human Lung Cell Atlas [24] (HLCA) as the reference set, a single-cell transcriptomic atlas of the human respiratory system encompassing data from 486 donors over 49 datasets. We noted a significant positive correlation between ECS% derived from HLCA deconvolution compared to HPCA deconvolution (Fig. 4, Pearson correlation 0.69, CI 0.67–0.71, p < 0.0001). ECS% as derived from the lung reference set when measured < 72 h from enrollment was significantly lower in survivors compared to non-survivors at 28 days (Median 2.0%, IQR 1.7–2.3% vs. 2.1% IQR 1.9–2.5% for non-survivors, p-value < 0.0001, Wilcoxon rank-sum).

**Fig. 5:**
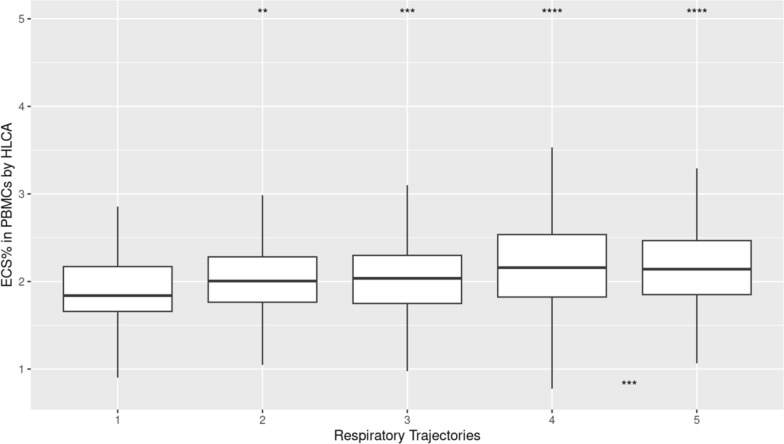
Higher percentage of EC signatures, as derived from the human lung dataset, were associated with worse respiratory trajectories of the IMPACC cohort. A modified ordinal score for oxygen requirements, based on the WHO scale, was recorded for all patients at all study timepoints. Based on these ordinal scales, five respiratory trajectories were previously described (Ozonoff, et. al) as represented in the x-axis. ECS % as determined by deconvolution based on the human lung dataset of PBMCs collected < 72 h tended to be higher in patients who developed worsening respiratory trajectories over the study (median 1.84%, IQR 1.66–2.17% for trajectory 1/with minimal requirements, 2.14%, IQR 1.85–2.20% for trajectory 5/with fatal outcome by day 28. Overall comparison for differences over all study days, One-way ANOVA, ***p < 0.001; Pair-wise comparisons with trajectory 1 as reference, Wilcoxon rank-sum, above each time point; **, p < 0.01, ***, p < 0.001, ****, p < 0.0001 n = 932)

We finally evaluated the distribution of the ECS% at < 72 h enrollment, as derived from the lung reference set, and compared the ranges between the different respiratory trajectories described above. We again found a statistically significant difference in < 72 h ECS% between patients with no oxygen requirements (trajectory 1) compared to those with worsening respiratory trajectories (median 1.8%, IQR 1.7–2.2% for trajectory 1/no requirements, 2.1%, IQR 1.9–2.2% for trajectory 5/with fatal outcome by day 28, p < 0.001, n = 932, one-way ANOVA, and p < 0.0001 for pairwise cox-regression between trajectories 1 and 5, Fig. 5).

As a second validation approach, given the lack of consensus on the appropriate protein signatures to be used in the enumeration of circulating endothelial cells, as a proof of principle, we leveraged data from other cells present in low frequency (5%) in the PBMC fraction that had well-defined protein signatures to evaluate the correlation between quantification outputs attained by deconvolution versus CYTOF [26]. Of the cells with measured CYTOF signatures in IMPACC that were also identified by deconvolution, plasmacytoid dendritic cells (DCs), CD8 + T cells, CD4 + T cells and B cells had deconvolution frequencies that had similarly low frequencies, comparable to endothelial cell signatures, for that particular cohort (plasmacytoid DCs: median 3.0% IQR 2.4–3.6%; CD8 + : median 4.1% IQR 2.1–6.3%; CD4 + : median 0.5% IQR 0.0–4.9%, and B Cells: 2.1%, IQR 0.5–4.0%, compared to ECS: median 2.5% IQR 2.1–2.9%). For all three cell types, there was a statistically significant Pearson correlation between the deconvolution and CYTOF outputs (Supplementary Table S2; plasmacytoid dendritic cells Pearson corr. 0.64, p < 0.0001; CD8 + cells Pearson corr. 0.62, p < 0.0001, CD4 + cells Pearson Corr. 0.66 p < 0.0001, B-cells Pearson corr. 0.70 p < 0.0001). In summary, for well characterized cell populations in circulation, deconvolution had a significant correlation to cells quantified by CYTOF, even when those populations were rare (< 5%).

## Discussion

Here we present novel findings related to the association of early endothelial cell transcriptomic signatures in the circulation of patients with ARDS or at risk for ARDS to worsening outcomes. The strength of this study is the inclusion of two very different cohorts- the validation cohort was clinically and demographically distinct from the pilot analysis, yet confirmed the general relationships found in our original analysis. CAF-PINT was almost fully composed of intubated patients, which enriched the initial analysis to more severe respiratory outcomes. IMPACC included a larger pool of patients early in the disease process and provided the opportunity of querying outcomes of a population at risk for respiratory failure, thus enabling the evaluation of the trajectory of respiratory illness over a full hospitalization. Moreover, the two cohorts covered a very wide age span- a pediatric population that included infants; and an adult population that included a geriatric population. As such, these findings support the implication that a rise in endothelial cell signature abundance (a surrogate for endothelial damage) is an important early event in worsening outcomes of respiratory failure in a varied population of patients.

The main limitation of this study is centered on the difficulty in defining the true signature of circulating endothelial cells- consensus in protein signature is lacking in the literature, while transcriptomic signatures are being extrapolated from two reference sets that received input of various endothelial cell types, which included tissue specific endothelial cells in monolayers in addition to suspended endothelial cells in culture, among others. Future studies need to integrate transcriptomics with endothelial-specific protein or imaging markers (e.g. CD31 + /CD146 + /CD45 −) and include single-cell RNA sequencing studies specifically designed to identify circulating endothelial cells as enriched from PBMC fractions [28] to further delineate precise transcriptomic signatures of CECs. Nevertheless, the GEDIT algorithm is a powerful tool that incorporates nuanced signatures of a variety of endothelial cells, and as such, may provide increased sensitivity for enumeration of these cells in circulation. Moreover, we indirectly tested the performance of the GEDIT algorithm by correlating the enumeration of well characterized low frequency cells by GEDIT (based on transcriptome) vs. CYTOF (based on surface protein markers) and found a significant correlation between experimental approaches, a surprising finding despite the differences among the assays (one measuring total RNA, the other a limited number of surface protein markers). It is well documented that not all mRNA ends up being expressed to protein level, and when they do, not all mRNA expresses as a readily assayed surface antigen. So it is particularly reassuring to identify a correlation in cell type enumeration despite translational shortcomings in the path between mRNA and protein, as these two assays are designed to measure. Finally, the fragility of CECs makes them especially vulnerable to manipulation, which is why GEDIT deconvolution, which analyses bulk RNA from peripheral blood in all conditions, may be a more sensitive approach to quantification of rare and delicate cell types.

Of note, the two cohorts had slight protocol differences in obtaining bulk RNA- CAFPINT captured the transcriptome of the whole blood collected in PAXgene tubes, IMPACC captured the transcriptome of PBMCs collected in CPT tubes. This likely affected the denominator by which percentages were calculated for each cell type, as whole blood may capture a wider variety of cell types. In practice, one can posit whether cells not in the PBMC population were sufficiently transcriptionally active to sway greater effect on the total population observed. Since we did not compare absolute numbers between cohorts and focused more on whether the results maintained similar relationships to outcomes, the consequence of these differences in methodology do not sway from the main findings. Regarding statistical approach, one limitation was that we did not correct for multiple comparisons, however we did find consistent associations in two independent cohorts.

In addition to two cohorts, we used two cell transcriptomic reference sets to validate our deconvolution data and mitigate concerns that any single reference set would bias our data to capture certain cell subtypes. We chose the Human Primary Cell Atlas (HPCA) as the reference set for the main analysis as it was most comprehensive and would capture the varied cells expected to be seen in circulation. Since the HPCA includes de-differentiated cell lines like HUVECs, which may decrease the specificity of the predicted endothelial cell signature, we included a reference set that exclusively incorporated tissue-specific RNA sequenced from lung samples. This dataset presumably increased the specificity to capture mature endothelial cells that were most likely to be in monolayers, while presumably losing sensitivity to capture untethered endothelial cells or those that were not of lung origin. The high degree of correlation between the output of two disparate reference datasets was remarkable, possibly reflecting substantial lung injury in IMPACC. Nevertheless, the observation that ECS% as estimated from each reference set had a conserved relationship to the primary and secondary outcomes enforced the validity of our approach.

To our knowledge, this was the first published study evaluating the trajectory of the abundance of the circulating EC signature over time. ECS% was noted to be elevated early after study recruitment. This was particularly remarkable for IMPACC, where some patients presented to the hospital with a COVID diagnosis but without respiratory failure. Given that the first IMPACC sample was collected within 72 h of enrollment, it is notable that even at this early time point, ECS% was associated with worsening respiratory trajectories and 28-day mortality. This perhaps supports an important pathophysiological role and potential prognostic strength of measuring endothelial damage as an early marker of mortality and respiratory outcomes in patients at risk for respiratory failure. Moreover, the plateauing of ECS% within 28 days for non-survivors (Fig. 1b) may support the utility of quantifying circulating endothelial signatures for prognostication in later time points once the opportunity for early detection has passed.

As it stands, utilizing bulk sequencing of blood followed by deconvolution to measure ECS% will likely not be a clinically viable means for prognostication in acutely ill patients- this would require identifying a parsimonious gene set that could be measured in real time, and that this gene set be further optimized to improve specificity of the signal to correspond to endothelial damage. Nevertheless, the findings described here raise the possibility that endothelial damage contributes to ARDS and may be sustained over many days. Indeed, the early increase in ECS% in circulation likely reflects both damaged ECs in circulation and potentially the innate response triggering other cells such as platelets, megakaryocytes, and leukocytes to express signals traditionally implicated in endothelial recruitment and transmigration of leukocytes to sites of injury, all of which reflect the central role of endothelial barrier loss to lung damage and dysfunction. The elevation in ECS% may also identify ARDS subgroups that are more susceptible to endothelial-targeted therapies, and even to identify therapeutic targets. Therefore, these findings emphasize the opportunity for prospective validation in independent, clinically diverse ARDS cohorts on how endothelial signatures in ARDS patients may represent early inciting events in the endothelial damage pathways of the lungs and other organs.

## Conclusion

Quantifying ECS abundance by deconvolution of bulk-RNA has supported a role for endothelial injury in respiratory failure in pediatric and adult patients at risk of worsening respiratory outcomes and mortality. Future studies may identify a parsimonious gene signature that can be applied for prognostic and predictive enrichment of patients at risk for ARDS.

## Supplementary Information

Additional file 1.

Additional file 2.

## Data Availability

IMPACC datafiles are available at ImmPort (www.immport.org, study ID SDY1760) and dbGAP accession number phs002686.v1.p1 (www.ncbi.nlm.nih.gov/gap/sstr/report/phs002686.v1.p1). For CAFPINT, all 740 peripheral blood RNA sequencing files are available in NIH dbGaP under accession ID phs003016.v1, (www.ncbi.nlm.nih.gov/gap/sstr/report/phs003016.v1.p1). The CAFPINT datasets supporting the conclusions of this article are available from the corresponding author on reasonable request.
