# Supplementary material for: Circulating endothelial signatures correlate with worse outcomes in COVID-19, respiratory failure and ARDS
Source: Crit Care. 2025 Oct 14;29:432. doi: 10.1186/s13054-025-05596-0 (PMC12522733; doi:10.1186/s13054-025-05596-0)
Supplement: Supplementary file 1 — Additional file 1. [file 13054_2025_5596_MOESM1_ESM.pdf]

Supplemental Materials

Methods:

Gene signatures

CAFPINT endothelial signature genes, HPCA

ADAMTS18	EFEMP1	IFI27	NTN4	SERPINE1
BMP4	EFNB2	KDR	PALMD	SHE
BMP6	EMCN	MMP1	PGF	SOX17
BMX	ERG	MMP10	PLSCR4	SOX7
CAV1	ESM1	MMRN1	PODXL	TFPI
CDH5	F2R	MMRN2	PROCR	TFPI2
CGNL1	FRY	MTUS1	PTPRB	TJP1
CLEC14A	GNG12	MYRIP	RGS5	TM4SF18
DOCK4	HHIP	NFIB	ROBO4	VWF
ECSCR	HMGA2	NR2F2	SERPIND1	ZNF521

IMPACC endothelial signature genes, HPCA

ADAMTS18	EFEMP1	IFI27	NTN4	SERPINE1
BMP4	EFNB2	KDR	PALMD	SHE
BMP6	EMCN	MMP1	PGF	SOX17
BMX	ERG	MMP10	PLSCR4	SOX7
CAV1	ESM1	MMRN1	PODXL	TFPI
CDH5	F2R	MMRN2	PROCR	TFPI2
CGNL1	FRY	MTUS1	PTPRB	TJP1
CLEC14A	GNG12	MYRIP	RGS5	TM4SF18
DOCK4	HHIP	NFIB	ROBO4	VWF
ECSCR	HMGA2	NR2F2	SERPIND1	ZNF521

CAFPINT endothelial signature genes, HLCA

ACVRL1	CLEC3B	FCN3	NOTCH4	SELP
ADGRL4	CYYR1	FOXF1	NPR3	SEMA3G
ARHGEF15	DACH1	JAM2	*	SHANK3
BTNL9	DLL4	JCAD	PCDH17	SLC6A4
CA4	EDN1	LDB2	PLVAP	SLCO2A1
CALCRL	EGFL7	LRRC32	PNMT	SOSTDC1
CCL14	EMCN	MMRN2	PRX	SOX18
CDH5	F2RL3	NDRG4	PTPRB	TEK
CLDN5	FAM107A	NES	RAMP3	TMEM100
CLEC14A	FAM167B	NOSTRIN	ROBO4	VWF

**of note, PCAT19 was not present in the CAFPINT dataset and as such only the top 49 gene signatures were used.*

IMPACC endothelial signature genes, HLCA

ACVRL1	CLEC3B	FCN3	NOTCH4	SELP
ADGRL4	CYYR1	FOXF1	NPR3	SEMA3G
ARHGEF15	DACH1	JAM2	PCAT19	SHANK3
BTNL9	DLL4	JCAD	PCDH17	SLC6A4
CA4	EDN1	LDB2	PLVAP	SLCO2A1
CALCRL	EGFL7	LRRC32	PNMT	SOSTDC1
CCL14	EMCN	MMRN2	PRX	SOX18
CDH5	F2RL3	NDRG4	PTPRB	TEK
CLDN5	FAM107A	NES	RAMP3	TMEM100
CLEC14A	FAM167B	NOSTRIN	ROBO4	VWF

RNA Sequencing methods

Briefly, for CAF-PINT, total RNA was extracted from whole blood using the PAXgene Blood RNA kit modified for pediatric use. Next, sequencing libraries were prepared using the Nugen universal plus kit with polyA capture and sequenced with the NovaSeq S4 system (Illumina) to generate 2 × 150 base paired-end reads to a target depth of 50 million read-pairs per sample. After quality control, 20,010 protein-coding genes were left for analysis. For IMPACC, RNA was extracted from PBMCs using the Quick-RNA MagBead Kit (Zymo). Next, sequencing libraries were prepared using SMART-Seq v4 Ultra Low Input RNA Kit (Takara Bio) and sequenced with the NovaSeq 6000 system (Illumina) to generate 2 × 100 base paired-end reads to a target depth of 25 million read-pairs per sample. Reads were trimmed for adapter sequence and quality score, aligned to a composite reference of human (GRCh38) reference sequence and SARS-CoV-2 (NCBI strain MN908947.3), and transcript abundance estimates were calculated. Of note, batch control analysis was previously performed for IMPACC[26] and no significant differences between batches were found.

Ethics

CAFPINT/HALFPINT: A data and safety monitoring board, whose members were appointed by the National Heart, Lung, and Blood Institute, monitored trial data and oversaw patients’ safety. Central ethics review was coordinated by the institutional review board at Boston Children’s Hospital, with appropriate signed institutional reliance agreements. A total of 10 study sites established reliance

relationships; at the remaining sites, oversight was conducted by a local institutional review board. Written informed consent was obtained from legal guardians, and assent was obtained from patients when appropriate.

IMPACC: NIAID staff conferred with the Department of Health and Human Services Office for Human Research Protections (OHRP) regarding the potential applicability of the public health surveillance exception [45CFR46.102(l) (2)] to the IMPACC study protocol. OHRP concurred that the study satisfied criteria for the public health surveillance exception, and the IMPACC study team sent the study protocol, and participant information sheet for review and assessment to institutional review boards (IRBs) at participating institutions. Twelve institutions elected to conduct the study as public health surveillance, while 3 sites with prior IRB-approved biobanking protocols elected to integrate and conduct IMPACC under their institutional protocols (University of Texas at Austin, IRB 2020-04-0117; University of California San Francisco, IRB 20-30497; Case Western Reserve University, IRB STUDY20200573) with informed consent requirements. Participants enrolled under the public health surveillance exclusion were provided information sheets describing the study, samples to be collected, and plans for data de-identification and use. Those who requested not to participate after reviewing the information sheet were not enrolled. In addition, participants did not receive compensation for study participation while inpatient, and subsequently were offered compensation during outpatient follow-ups.

Supplemental Results

Per reviewer request, we have added information on COVID variants, the data we had on steroid and immunosuppressive therapies, and VTE outcomes.

COVID Variants in IMPACC

Partly because of how early in the pandemic most patients were enrolled, and also due to lack of sequences for many patients, the data on variants wasn’t particularly insightful for IMPACC. Viral sequencing, available in less than half of the cohort,

revealed few variants of concern or interest that circulated during the study conduct [7 patients with alpha (B.1.1.7), 12 with epsilon (B.1.427 and B.1.429) and 4 with iota (B1.429)]. Our cohort was also fully enrolled prior to the widespread circulation of the SARS-CoV-2 B.1.617.2 (Delta), and B.1.1.529 (Omicron) variants which are associated with higher transmission, and possibly different severity of COVID-19[1].

Association of ECS% and treatment modalities in IMPACC.

Due to the timing of the IMPACC dataset, which predated most formal recommendations on Remdesivir and Dexamethasone use, the IMPACC cohort data includes binary data on whether patients ever received remdesivir, steroids (with no data on which type or dosage) and other immunosuppressive agents, without any information on timing in relation to sample collection. Given this, about 61% of the cohort received remdesivir, 67% steroids, 6% other immunosuppression. We observed no difference in ECS % at first collection between patients who ever received remdesivir vs those who did not (P=0.374) or immunosuppression (P=0.381), but ECS% was higher in patients who ever received steroids compared to those who did not (P=0.001, plot below).

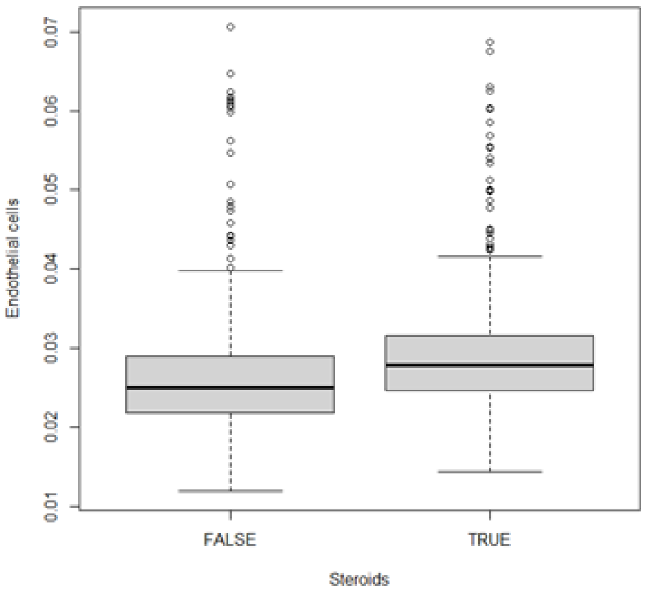

We also performed analysis of steroids split by day-28 mortality. The highest ECS% was observed for non-survivors treated with steroids (P=0.0001) or without steroids (P=0.008), but was also notably higher in patients who survived with steroids (P=0.001) compared to survivors not receiving steroids.

[1] IMPACC Manuscript Writing IMPACC Manuscript Writing Team et al., "Immunophenotyping Assessment in a COVID-19 Cohort (IMPACC): A Prospective Longitudinal Study.," *Science Immunology* 6, no. 62 (August 10, 2021), https://doi.org/10.1126/sciimmunol.abf3733.

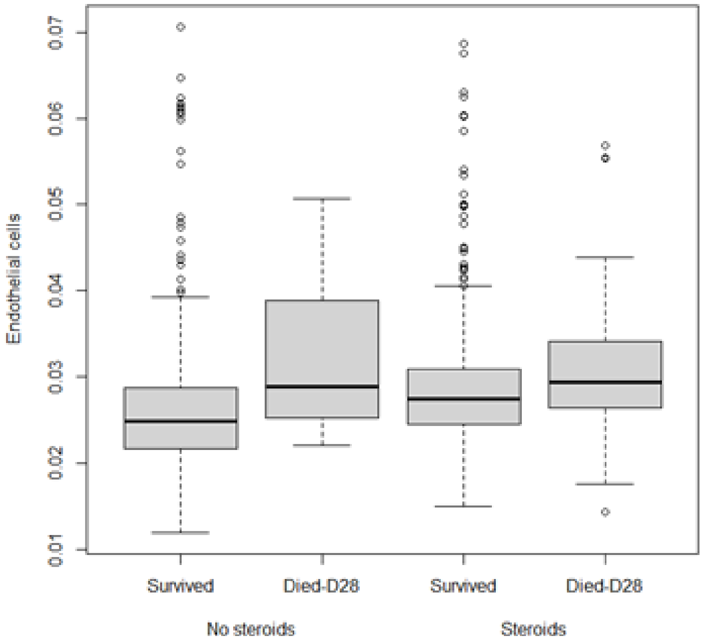

However, in interaction term analysis, we found that steroid treatment did not interact with the association between ECS% and mortality (P= 0.8).

Association of ECS% and the Outcome of Vascular Thrombotic Events in IMPACC.

Out of >1000 IMPACC patients, there were 70 reported cases of VTE in this cohort. We observed that ECS% at first collection was not significantly different between patients who had VTE compared to those without VTE (p=0.541), though the cohort may not have been appropriately powered to evaluate this.

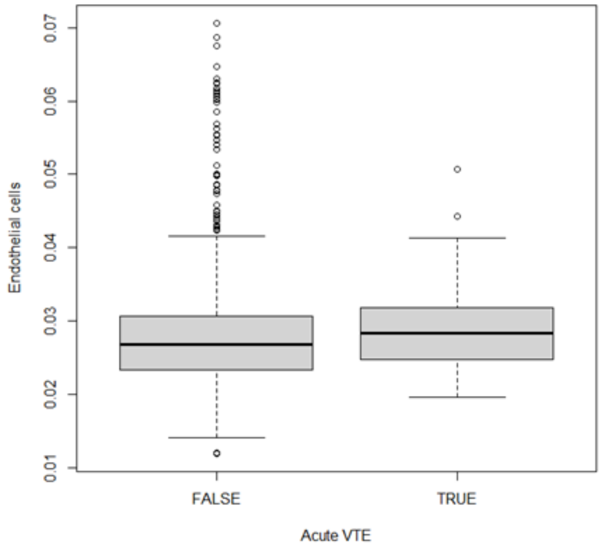

Comparison of Predicted Enumeration of Rare Cells Between Deconvolution and CyTOF methods-sample scatterplots.

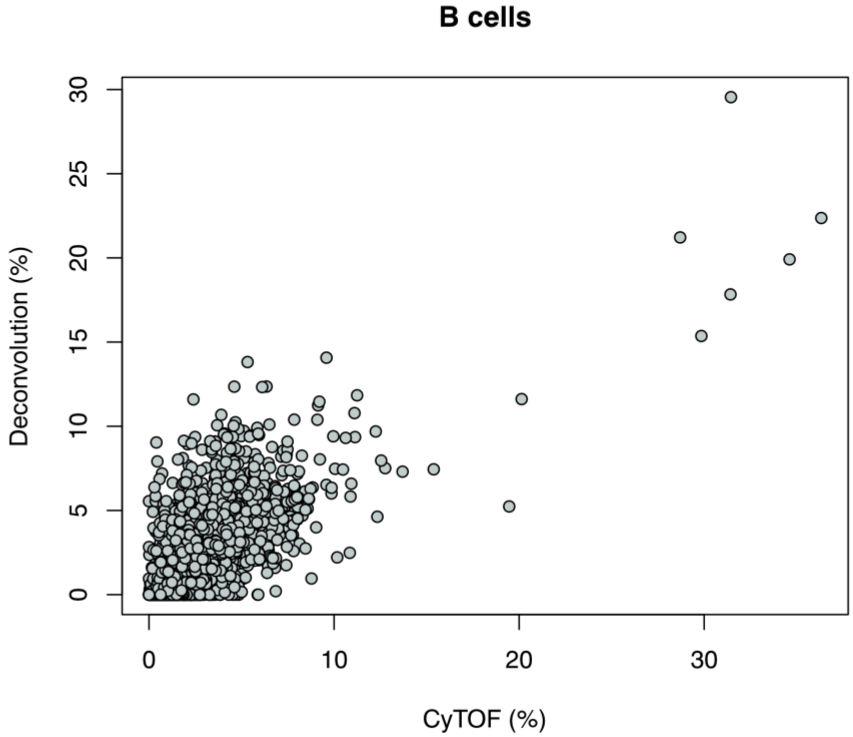

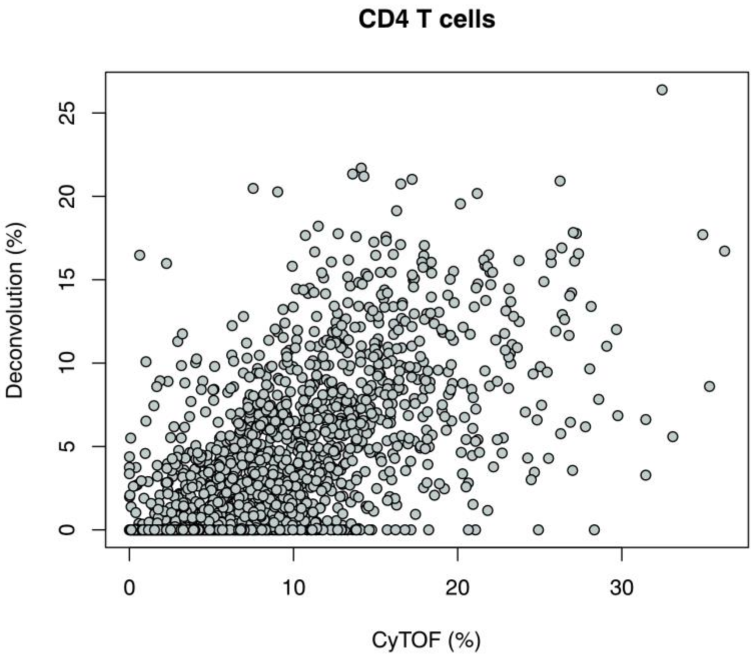
CD4 T cells
Deconvolution (%)
CyTOF (%)
0
5
10
15
20
25
0
10
20
30

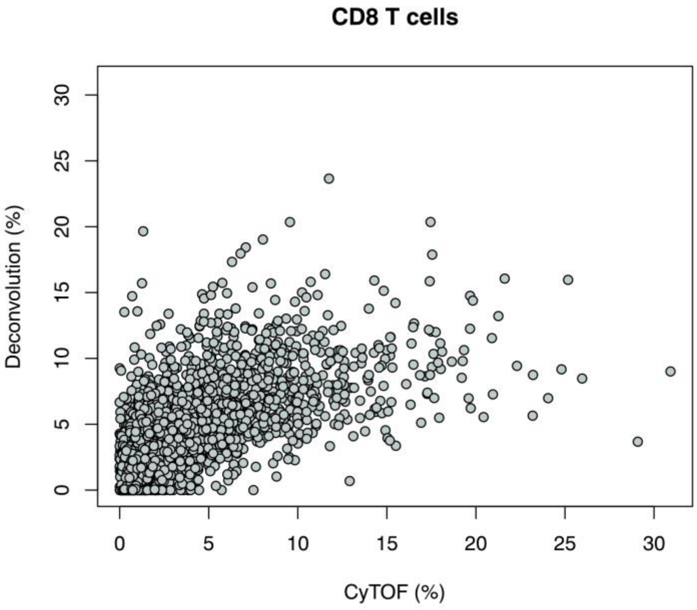
CD8 T cells
Deconvolution (%)
CyTOF (%)
0
5
10
15
20
25
30
0
5
10
15
20
25
30
