# Supplementary material for: Circulating endothelial signatures correlate with worse outcomes in COVID-19, respiratory failure and ARDS
Source: Crit Care. 2025 Oct 14;29:432. doi: 10.1186/s13054-025-05596-0 (PMC12522733; doi:10.1186/s13054-025-05596-0)
Supplement: Supplementary file 2 — Additional file 2. [file 13054_2025_5596_MOESM2_ESM.docx]

*Table S1- CAFPINT Development of PARDS at any time during hospitalization*

|  | OR | CI | P value |
| --- | --- | --- | --- |
| Univariable (no adjustments) | | | |
| ECS % | 2.07 | 1.16-3.88 | <0.05 |
|  | | | |
| Multivariable (adjusted for sex, race and ethnicity) | | | |
| ECS % | 2.19 | 1.20-4.29 | <0.05 |
| PRISM Score | 1.02 | 0.97-1.07 | NS |
| Age (Years) | 0.91 | 0.84-0.97 | <0.01 |

*Table S2- Comparison between GEDIT deconvolution and CYTOF outputs for low frequency cell populations*

| ***Cell Type*** | ***Pearson Correlation***  ***Coefficient*** | ***Pearson***  ***Correlation p-value*** | ***Lme***  ***Coef*** | ***Lme***  ***p-value*** | ***Spearman Correlation***  ***Coefficient*** | ***Spearman***  ***Correlation p-value*** |
| --- | --- | --- | --- | --- | --- | --- |
| *B Cells* | *0.70* | *<0.0001* | *0.58* | *<0.0001* | *0.45* | *<0.0001* |
| *CD4+ T Cells* | *0.66* | *<0.0001* | *0.39* | *<0.0001* | *0.51* | *<0.0001* |
| *CD8+ T Cells* | *0.62* | *<0.0001* | *0.43* | *<0.0001* | *0.45* | *<0.0001* |
| *Plasmacytoid DCs* | *0.64* | *<0.0001* | *0.54* | *<0.0001* | *0.18* | *<0.0001* |
